# Comparative efficacy of generic nifedipine versus brand‐name amlodipine for hypertension management in Taiwan

**DOI:** 10.1111/jch.14521

**Published:** 2022-06-08

**Authors:** Hao‐Wei Lee, Chin‐Chou Huang, Hsin‐Bang Leu, Yenn‐Jiang Lin

**Affiliations:** ^1^ Division of Cardiology Department of Medicine Taipei Veterans General Hospital Taipei Taiwan; ^2^ School of Medicine National Yang Ming Chiao Tung University Taipei Taiwan; ^3^ Institute of Pharmacology School of Medicine National Yang Ming Chiao Tung University Taipei Taiwan; ^4^ Cardiovascular Research Center National Yang Ming Chiao Tung University Taipei Taiwan; ^5^ Healthcare and Services Center Taipei Veterans General Hospital Taipei Taiwan; ^6^ Heart Rhythm Center Taipei Veterans General Hospital Taipei Taiwan

**Keywords:** amlodipine, generic, hypertension, nifedipine, outcome

## Abstract

The control rate of hypertension remains concerning, indicating the requirement for better management strategies. The calcium channel blockers brand‐name amlodipine and nifedipine with extended‐release formulations demonstrate similar clinical efficacy. However, the efficacy of generic nifedipine remains obscure. We compared the efficacy of generic nifedipine and brand‐name amlodipine in terms of cardiovascular (CV) outcomes. Patients prescribed generic nifedipine (SRFC CYH) or brand‐name amlodipine besylate (Norvasc, Pfizer) between August 1, 2017, and July 31, 2018, were enrolled; patients with CV events within 3 months were excluded. CV outcomes included CV death, nonfatal myocardial infarction (MI), nonfatal ischemic stroke, hospitalization for heart failure, and composite endpoints of 3P‐ and 4P‐major adverse cardiac events (MACE). **A** total of 1625 patients treated with nifedipine (SRFC CYH) and 16 587 patients treated with Norvasc were included. After propensity score matching, there were 995 and 4975 patients in the nifedipine CYH and Norvasc groups, respectively. At a mean follow‐up period of 30.3 ± 6.4 months, nifedipine CYH was comparable to Norvasc in terms of CV death (*P *= .107), nonfatal MI (*P *= .121), nonfatal ischemic stroke (*P *= .453), hospitalization for heart failure (*P *= .330), 3P‐MACE (*P *= .584), and 4P‐MACE (*P *= .274). Cox regression analysis revealed that nifedipine CYH and Norvasc had similar efficacy in terms of 3P‐MACE (hazard ratio, 0.970; 95% confidence interval, 0.601–1.565, *P *= .900) and 4P‐MACE (hazard ratio, 0.880; 95% confidence interval, 0.628–1.233, *P *= .459). In conclusion, Nifedipine SRFC CYH and Norvasc have comparable clinical efficacy for hypertension management.

## 1 INTRODUCTION

Hypertension is one of the most predominant risk factors for cardiovascular (CV) diseases and is a global public health challenge.^1^, ^2^ Despite improvements in the awareness, treatment, and control of hypertension in the past few years, the control rate of hypertension is concerning,^3^, ^4^ implying that more efficient antihypertensive strategies are required.

Both nifedipine and amlodipine are widely used dihydropyridine calcium‐channel blockers (CCBs) for hypertension management.^5^, ^6^, ^7^, ^8^ The development of extended‐release formulations improved the safety profile of the original short‐acting nifedipine.^9^, ^10^ Fiscal outcomes compared with both the gastrointestinal therapeutic system (GITS) and the osmotic‐controlled release oral delivery system (OROS) of nifedipine use osmotic pressure as the driving force to deliver drugs through laser‐drilled holes, providing stable drug concentrations, uniform drug effect, and reduced dosing frequency.^11^, ^12^ Furthermore, with regard to clinical efficacy, application of extended‐release formulations of nifedipine demonstrated similar blood pressure (BP)‐lowering effects as long‐acting amlodipine.^13^, ^14^

In contrast, because of their lower costs compared to their brand‐name counterparts, generic drugs are commonly used in the health care system globally. Over the past decade, about 90% of prescribed medications in the United States were generics, which only accounted for 26% of drug expenditure, saving approximately $1.7 trillion dollars.^15^ Generic drugs have the same active chemical ingredients as brand‐name products; however, whether the pharmaceutical equivalence and bioequivalence between both of them translate to evenly matched clinical outcomes remain unclear.

In 2018, owing to the decrease in the drug production capacity of the parent manufacturing company, the import of the brand‐name nifedipine (Adalat OROS, Bayer) to Taiwan was greatly impacted, and the supply chain was not completely restored until 2021. Owing to the shortage of Adalat, generic nifedipine was used as an alternative. However, the clinical efficacy of generic nifedipine remains unknown. Recently, a nationwide study in Taiwan revealed comparable clinical outcomes between generic nifedipine and its brand‐name counterpart.^16^ In this study, we aimed to compare the efficacy of a generic nifedipine with brand‐name amlodipine in terms of CV outcomes.

## 2 METHODS

### 2.1 Study participants

The study was conducted in the outpatient clinics of Taipei Veterans General Hospital, a national medical center in Taiwan. All patients were enrolled if they met the following criteria: (1) age ≥ 20 years, (2) clinically diagnosed hypertension, and (3) received either generic nifedipine (Nifedipine SRFC, Chunghwa Yuming Healthcare Co., Taiwan) or brand‐name amlodipine besylate (Norvasc, Pfizer)^17^ between August 1, 2017, and July 31, 2018. Patients with CV events within 3 months were excluded.

The study protocol was approved by the Ethics Committee of the Taipei Veterans General Hospital (approval number: 2020‐09‐012BC). The requirement for informed consent was waived because of the retrospective nature of the study. This study was conducted in accordance with the principles of the Declaration of Helsinki.

### 2.2 Study design

**FIGURE 1:**
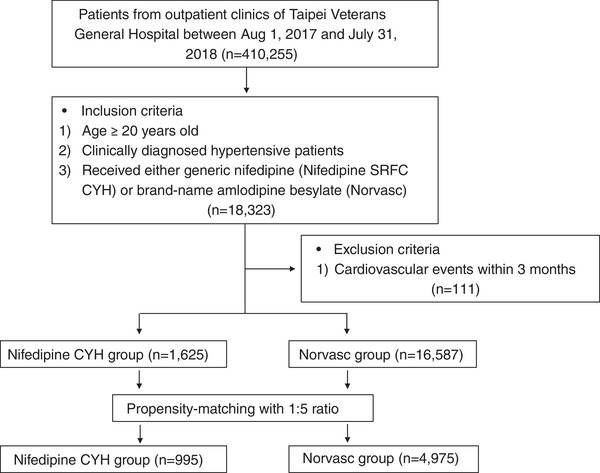
Study flowchart

The flowchart of the study is presented in Figure 1. This was a retrospective study. All the data were collected from the Taipei Veterans General Hospital database. The baseline characteristics, including office BPs, comorbidities, and laboratory data, were collected. Office BPs were defined as the BPs after generic nifedipine or brand‐name amlodipine for at least 2 weeks. Comorbidities were defined according to the ICD‐9 and ICD‐10 codes, which included diabetes mellitus (DM), chronic kidney disease (CKD), heart failure, arrhythmia, and hyperlipidemia, and a history of myocardial infarction (MI), ischemic stroke, and hemorrhagic stroke.

Hypertensive patients who used nifedipine (SRFC; Chunghwa Yuming Healthcare Co., Taiwan) for ≥3 months were selected as the nifedipine CYH group. Patients with hypertension who used amlodipine besylate (Norvasc, Pfizer) for ≥3 months were included in the control group.

### 2.3 Clinical outcomes

The index date was defined as the date of any event or the date of last follow up. The clinical outcomes of the study included CV death, nonfatal MI, nonfatal ischemic stroke, heart failure hospitalization, and the composite endpoints of 3P‐major adverse cardiac events (MACE) and 4P‐MACE. 3P‐MACE were defined as the composite endpoints of CV death, nonfatal MI, and nonfatal stroke. 4P‐MACE were defined as the composite endpoints of CV death, nonfatal MI, nonfatal stroke, and heart failure hospitalization.

### 2.4 Statistical analyses

The characteristics of the participants were summarized using descriptive statistics. Quantitative variables are expressed as mean ± standard deviation, and categorical variables are expressed as numbers (percentages). Parametric continuous data between hypertensive patients in the nifedipine CYH and control groups were compared using the unpaired Student's t‐test, and nonparametric data were compared using the Mann–Whitney test.

To minimize the possible confounding factors, we created a propensity model to compare clinical outcomes between the two groups. The model included age, sex, office systolic blood pressure (SBP), office diastolic blood pressure (DBP), heart rate, and comorbidities. Patients in the nifedipine CYH group were then matched with those in the control group at a 1:5 ratio using the propensity‐matching algorithm. Parametric continuous data between hypertensive patients in the nifedipine CYH and control groups were compared using the unpaired Student's t‐test, and nonparametric data were compared using the Mann–Whitney test.

Survival analysis was performed using the Kaplan–Meier curve, with significance based on the log‐rank test. Cox proportional hazards regression analysis was performed. Adjusted hazard ratios (HRs) with 95% confidence intervals (CIs) were estimated after adjusting for the potential confounding factors.

Statistical significance was inferred at a two‐sided *P‐value* < .05. Statistical analysis was performed using the SPSS software (version 21.0, SPSS Inc., Chicago, IL, USA).

## 3 RESULTS

### 3.1 Demographic data of the participants

A total of 410 255 patients had visited the outpatient clinics of the Taipei Veterans General Hospital between August 1, 2017, and July 31, 2018. Among them, 1625 hypertensive patients receiving nifedipine CYH and 16 587 hypertensive patients receiving Norvasc fulfilled the inclusion criteria. Compared to the Norvasc group, the nifedipine CYH group consisted of patients who were younger (*P *= .001) and predominantly male (*P *< .001); had higher SBP (*P *< .001) and DBP (*P *= .024), higher creatinine levels (*P *< .001), lower estimated glomerular filtration rate (*P *< .001), and lower alanine aminotransferase levels (*P *= .010). Furthermore, the nifedipine CYH group had more cases of DM (*P *< .001), CKD (*P *< .001), and heart failure (*P *= .005) and fewer cases with a history of hemorrhagic stroke (*P *< .001) than the Norvasc group. Patients in the nifedipine CYH group used more concomitant antihypertensive agents (*P *< .001), including ACEI/ARB (*P* < .001), β‐blocker (*P* < .001, and thiazide diuretics (*P* < .001). There were more statins use in patients in the nifedipine CYH group (*P* < .001) (Table 1).

**TABLE 1:** Baseline characteristics

	Nifedipine CYH (no. = 1625)	Norvasc (no. = 16 587)	*P*
Age, years	68.2 ± 14.2	69.4 ± 13.5	.001
Male, no. (%)	960 (59.1%)	8912 (53.7%)	<.001
SBP, mmHg	144.9 ± 22.6	139.3 ± 20.0	<.001
DBP, mmHg	76.1 ± 13.9	75.2 ± 12.8	.024
Heart rate, bpm	78.0 ± 13.0	78.2 ± 13.7	.576
DM, no. (%)	573 (35.3%)	4428 (26.7%)	<.001
CKD, no. (%)	286 (17.6%)	734 (4.4%)	<.001
Heart failure, no. (%)	65 (4.0%)	461 (2.8%)	.005
Arrhythmia, no. (%)	119 (7.3%)	1331 (8.0%)	.319
Atrial fibrillation, no. (%)	44 (2.7%)	528 (3.2%)	.294
Hyperlipidemia, no. (%)	499 (30.7%)	5039 (30.4%)	.784
History of MI, no. (%)	14 (0.9%)	115 (0.7%)	.440
History of ischemic stroke, no. (%)	49 (3.0%)	549 (3.3%)	.525
History of hemorrhagic stroke, no. (%)	14 (0.9%)	366 (2.2%)	<.001
Total cholesterol, mg/dL	177.6 ± 40.1	179.1 ± 37.5	.228
LDL‐C, mg/dL	102.5 ± 32.0	103.8 ± 31.8	.165
Creatinine, mg/dL	2.0 ± 2.4	1.2 ± 1.2	<.001
eGFR, mL/min/1.73 m^2^	56.1 ± 27.3	68.2 ± 22.2	<.001
ALT, U/L	24.4 ± 14.7	25.7 ± 14.9	.010
ACEI/ARB, no. (%)	1,019 (62.7%)	7,935 (47.8%)	<.001
β‐blocker, no. (%)	828 (51.0%)	4,949 (29.8%)	<.001
Thiazide diuretics, no. (%)	280 (17.2%)	1,892 (11.4%)	<.001
Number of other antihypertensive drugs, no. (%)	1.3 ± 0.9	0.9 ± 0.9	<.001
Statins, no. (%)	646 (39.8%)	5,818 (35.1%)	<.001

Abbreviations: ACEI, angiotensin‐converting enzyme inhibitor; ALT, alanine aminotransferase; ARB, angiotensin receptor blockers; CKD, chronic kidney disease; DBP, diastolic blood pressure; DM, diabetes mellitus; eGFR, estimated glomerular filtration rate; LDL‐C, low‐density lipoprotein cholesterol; MI, myocardial infarction; SBP, systolic blood pressure.

After propensity matching, 995 hypertensive patients receiving nifedipine CYH and 4975 hypertensive patients receiving Norvasc were selected for the study. A total of 57 patients in the Nifedipine CYH group (5.7%) had ever used Norvasc in the follow‐up period, while they did not change the groups. Most of the characteristics were similar between the two groups, while the patients in the nifedipine CYH group used more concomitant antihypertensive agents (*P* < .001), including ACEI/ARB (*P* < .001), β‐blocker (*P* < .001), and thiazide diuretics (*P* < .001) (Table 2).

**TABLE 2:** Baseline characteristics (propensity‐matched)

	Nifedipine CYH (no. = 995)	Norvasc (no. = 4975)	*P*
Age, years	69.1 ± 13.8	69.6 ± 13.4	.281
Male, no. (%)	588 (59.1%)	2876 (57.8%)	.453
SBP, mmHg	141.4 ± 20.6	141.6 ± 20.6	.802
DBP, mmHg	75.9 ± 13.9	75.5 ± 13.2	.380
Heart rate, bpm	77.7 ± 13.1	78.3 ± 13.8	.200
DM, no. (%)	353 (35.5%)	1760 (35.4%)	.952
CKD, no. (%)	64 (6.4%)	300 (6.0%)	.629
Heart failure, no. (%)	34 (3.4%)	173 (3.5%)	.924
Arrhythmia, no. (%)	84 (8.4%)	411 (8.3%)	.850
Atrial fibrillation, no. (%)	28 (2.8%)	177 (3.6%)	.240
Hyperlipidemia, no. (%)	341 (34.3%)	1698 (34.1%)	.932
History of MI, no. (%)	8 (0.8%)	37 (0.7%)	.841
History of ischemic stroke, no. (%)	33 (3.3%)	167 (3.4%)	.949
History of hemorrhagic stroke, no. (%)	9 (0.9%)	47 (0.9%)	.904
Total cholesterol, mg/dL	177.0 ± 38.9	177.8 ± 38.2	.590
LDL‐C, mg/dL	102.7 ± 31.4	102.1 ± 31.9	.638
Creatinine, mg/dL	1.3 ± 1.4	1.3 ± 1.2	.581
eGFR, mL/min/1.73m^2^	64.2 ± 22.7	63.8 ± 22.5	.668
ALT, U/L	25.0 ± 14.7	25.2 ± 14.6	.777
ACEI/ARB, no. (%)	664 (66.7%)	2650 (53.3%)	<.001
β‐blocker, no. (%)	471 (47.3%)	1669 (33.5%)	<.001
Thiazide diuretics, no. (%)	182 (18.3%)	635 (12.8%)	<.001
Number of other antihypertensive drugs, no. (%)	1.3 ± 0.9	1.0 ± 0.9	<.001
Statins, no. (%)	448 (45.0%)	2112 (42.5%)	.134

Abbreviations: ACEI, angiotensin converting enzyme inhibitor; ALT, alanine aminotransferase; ARB, angiotensin receptor blockers; CKD, chronic kidney disease; DBP, diastolic blood pressure; DM, diabetes mellitus; eGFR, estimated glomerular filtration rate; LDL‐C, low‐density lipoprotein cholesterol; MI, myocardial infarction; SBP, systolic blood pressure.

### 3.2 Clinical outcomes of the participants

During 30.3 ± 6.4 months of follow‐up, there were 25 CV deaths, 28 nonfatal MIs, 81 nonfatal ischemic strokes, and 146 heart failure hospitalizations. Among them, there were similar rates of CV death (nifedipine CYH vs Norvasc = 0.1% vs 0.5%, *P *= .107), nonfatal MI (nifedipine CYH vs. Norvasc = 0.8% vs 0.4%, *P *= .121), nonfatal ischemic stroke (nifedipine CYH vs Norvasc = 1.1% vs. 1.4%, *P* = .453), heart failure hospitalization (nifedipine CYH vs Norvasc = 2.0% vs 2.5%, *P* = .330), 3P‐MACE (nifedipine CYH vs Norvasc = 2.0% vs 2.3%, *P* = .584), and 4P‐MACE (nifedipine CYH vs Norvasc = 4.0% vs 4.8%, *P* = .274) in the two groups (Table 3).

**TABLE 3:** Cardiovascular outcomes (propensity‐matched)

	Nifedipine CYH(no. = 995)	Norvasc (no. = 4975)	*P*
CV death, no. (%)	1 (0.1%)	24 (0.5%)	.107
Nonfatal MI, no. (%)	8 (0.8%)	20 (0.4%)	.121
Nonfatal ischemic stroke, no.(%)	11 (1.1%)	70 (1.4%)	.453
Heart failure hospitalization, no.(%)	20 (2.0%)	126 (2.5%)	.330
3P‐MACE, no.(%)	20 (2.0%)	114 (2.3%)	.584
4P‐MACE, no.(%)	40 (4.0%)	240 (4.8%)	.274

Abbreviations: CV, cardiovascular; MACE, major adverse cardiovascular event; MI, myocardial infarction.

3P‐MACE: CV death, nonfatal MI, nonfatal ischemic stroke.

4P‐MACE: CV death, nonfatal MI, nonfatal ischemic stroke, and heart failure hospitalization.

**FIGURE 2:**
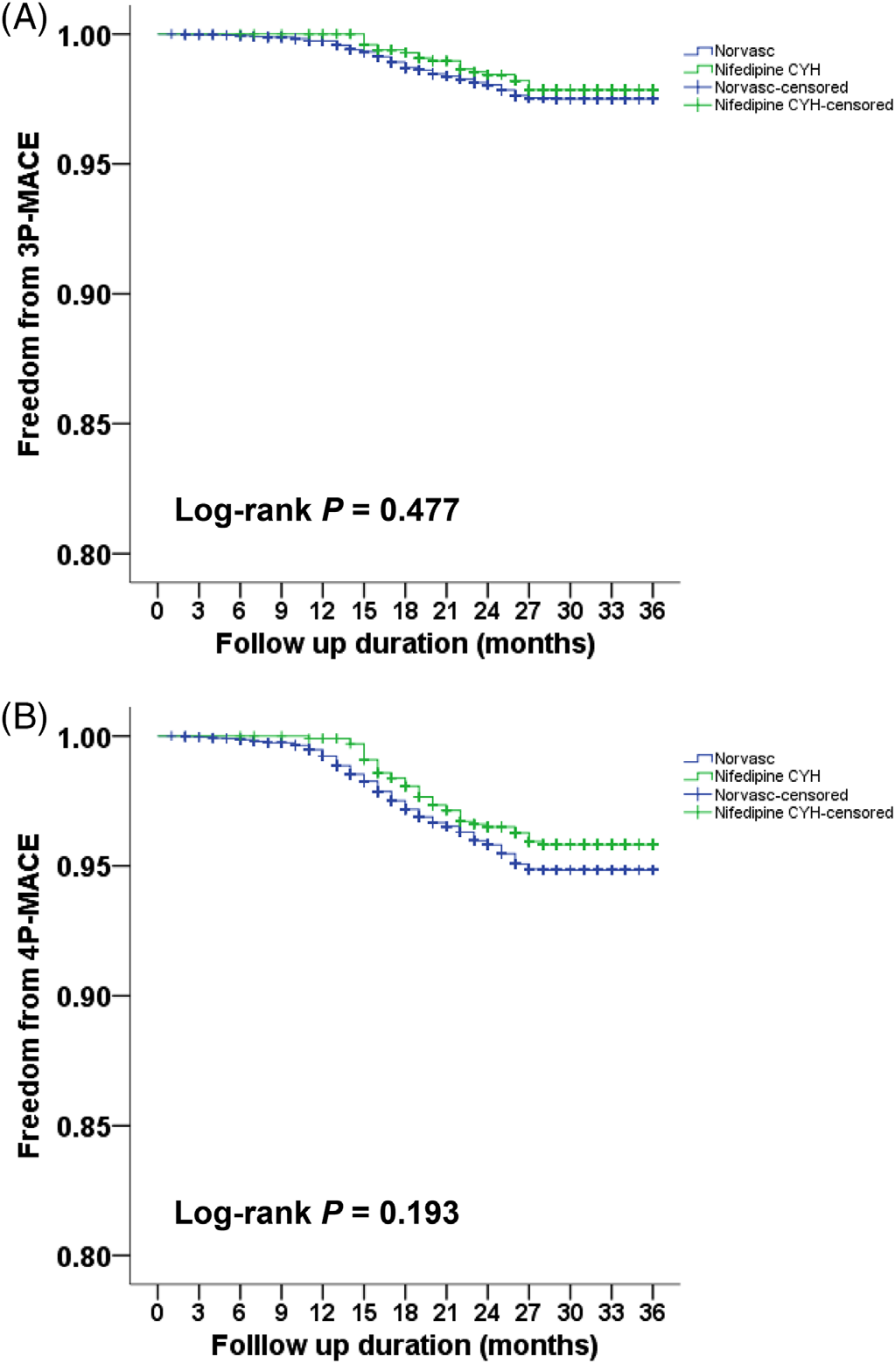
Kaplan–Meier survival curves showing the absence of major adverse cardiovascular events (MACE) according to the use of a calcium channel blocker in patients with hypertension. The blue line represents the patient group administered Norvasc. The green line represents the group administered nifedipine CYH. Differences were compared using the log‐rank test. (A) 3P‐MACE, defined as the composite endpoint of cardiovascular death, nonfatal myocardial infarction, and nonfatal stroke (*P *= .477). (B) 4P‐MACE, defined as the composite endpoint of cardiovascular death, nonfatal myocardial infarction, nonfatal stroke, and heart failure hospitalization (*P *= .193)

Kaplan–Meier survival curves and log‐rank tests were used to compare the number of patients who did not exhibit any clinical CV event during the follow‐up period. The incidences of 3P‐MACE (*P* = .477, Figure 2A) and 4P‐MACE (*P* = .193, Figure 2B) were similar between the two groups.

Univariate Cox regression analysis revealed that nifedipine CYH had similar efficacy as Norvasc in 3P‐MACE (HR, 0.842; 95% CI, 0.524–1.354, *P* = .478; Table 4) and 4P‐MACE (HR, 0.801; 95% CI, 0.573–1.120, *P* = .194; Table 5).

**TABLE 4:** Cox regression for 3P‐MACE (propensity‐matched)

	HR	95% CI	*P*
Univariate analysis			
Nifedipine CYH (yes vs no)	0.842	(0.524 – 1.354)	.478
Multivariate analysis			
Nifedipine CYH (yes vs no)	0.970	(0.601 – 1.565)	.900
Age, years	1.028	(1.015 – 1.042)	<.001
Male (yes vs no)	1.482	(1.032 – 2.127)	.033
ACEI/ARB (yes vs no)	0.442	(0.302 – 0.646)	<.001
β‐blocker (yes vs no)	0.869	(0.595 – 1.268)	.465
Thiazide diuretics (yes vs no)	0.818	(0.427 – 1.566)	.544
Statins (yes vs no)	0.453	(0.302 – 0.678)	<.001

Abbreviations: ACEI, angiotensin‐converting enzyme inhibitor; ARB, angiotensin receptor blockers; CI, confidence interval; HR, hazard ratio; MACE, major adverse cardiovascular event.

3P‐MACE: CV death, nonfatal MI, nonfatal ischemic stroke.

**TABLE 5:** Cox regression for 4P‐MACE (propensity‐matched)

	HR	95% CI	*P*
Univariate analysis			
Nifedipine CYH (yes vs no)	0.801	(0.573 – 1.120)	.194
Multivariate analysis			
Nifedipine CYH (yes vs no)	0.880	(0.628 – 1.233)	.459
Age, years	1.039	(1.029 – 1.049)	<.001
Male (yes vs no)	1.205	(0.945 – 1.536)	.132
ACEI/ARB (yes vs no)	0.481	(0.371 – 0.623)	<.001
β‐blocker (yes vs no)	1.179	(0.920 – 1.512)	.194
Thiazide diuretics (yes vs no)	0.856	(0.559 – 1.311)	.475
Statins (yes vs no)	0.484	(0.369 – 0.636)	<.001

Abbreviations: ACEI, angiotensin‐converting enzyme inhibitor; ARB, angiotensin receptor blockers; CI, confidence interval; HR, hazard ratio; MACE, major adverse cardiovascular event.

4P‐MACE: CV death, nonfatal MI, nonfatal ischemic stroke, and heart failure hospitalization.

Multivariate Cox regression analysis revealed consistent data that nifedipine CYH had similar efficacy as Norvasc in 3P‐MACE (HR, 0.970; 95% CI, 0.601–1.565, *P* = .900; Table 4) and 4P‐MACE (HR, 0.880; 95% CI, 0.628–1.233, *P* = .459; Table 5).

## 4 DISCUSSION

This retrospective study compared the clinical outcomes of hypertensive patients treated with SRFC CYH and those treated with Norvasc. We found that the efficacy of SRFC CYH was similar to that of Norvasc in terms of CV death, nonfatal MI, nonfatal ischemic stroke, heart failure hospitalization, and composite CV outcomes.

### 4.1 Generic CCB versus brand‐name CCB

Earlier studies have suggested that generic antihypertensive medications may be as effective as brand‐name drugs for managing BP. In a systematic review and meta‐analysis comprising 47 studies, seven randomized controlled trials (RCTs) focusing on generic and brand‐name CCBs used to manage CV diseases were evaluated. The results showed that the BP‐lowering effect of generic CCBs was similar to that of brand‐name CCBs.^18^ Another meta‐analysis of 74 RCTs, with seven RCTs focusing on CCB, revealed no remarkable differences between generic and brand‐name drugs with regard to efficacy and adverse events.^19^ Nevertheless, these trials were limited by their short follow‐up periods, small sample sizes, and inclusion of disproportionately young and healthy participants. Moreover, most of these studies on CCBs investigated only amlodipine, whereas a few investigated nondihydropyridine CCBs, thereby leaving nifedipine unstudied.

Recently, the long‐term clinical outcomes of generic nifedipine (SRFC CYH) and brand‐name nifedipine (Adalat OROS, Bayer) were compared in a nationwide, retrospective, cohort study involving 98 335 patients with hypertension in the National Health Insurance Research Database, Taiwan. At a mean follow‐up of 4.1 years, the study revealed similar effects of the two drugs—generic (n = 21 087) and brand‐name (n = 77 248) nifedipine—in terms of all‐cause mortality (7.2% vs 7.1%; HR, 1.02; 95% CI, 0.95–1.09, *P *= .597) and composite CV outcome (11.6% vs 11.9%; HR, 0.97; 95% CI, 0.92–1.03, *P* = .354), including CV death, nonfatal MI, nonfatal stroke, coronary revascularization, and heart failure hospitalization. Despite the fact that increased rates of headache, peripheral edema, and constipation were observed in the generic group, the efficacy of generic nifedipine was not inferior to that of its brand‐name counterpart.^16^

### 4.2 Short‐acting CCBs with extended‐release formulations versus long‐acting CCBs

Short‐acting CCBs with extended‐release formulations have been developed to maintain BP control, as patients using short‐acting CCBs may commonly experience uncontrolled hypertension due to suboptimal compliance, especially during the initial stage of treatment.^11^, ^12^ Short‐acting CCBs with extended‐release formulations displayed more uniform and lasting drug effects than the prototype. An RCT using an arterial line for BP monitoring demonstrated that extended‐release nifedipine (nifedipine GITS; n = 15) provided a more constant drug concentration than short‐acting nifedipine (n = 16; *P* < .001) and placebo (n = 9). Moreover, extended release of nifedipine led to a persistent decrease in BP over 360 minutes (*P* = .0028), whereas the BP‐lowering effect of short‐acting nifedipine only remained significant through the 300‐minute timepoint (*P* = .0028). A significant difference was noted in the change in mean arterial BP over time among the three treatment groups (*P* < .001).^20^

### 4.3 Short‐acting nifedipine with extended‐release formulations versus amlodipine

Comparisons were made between the BP‐lowering effects of short‐acting nifedipine with an extended‐release formulation and long‐acting CCB, namely, amlodipine. Nonetheless, previous studies evaluated a relatively small number of patients and were conducted solely in clinics and did not conduct ambulatory BP monitoring.^20^, ^21^, ^22^, ^23^, ^24^

Recently, the BP‐lowering effect of nifedipine GITS and amlodipine as monotherapy has been tested in an RCT on hypertensive patients without a history MI or stroke in the past 2 years before enrollment. The study demonstrated similar BP reduction after treatment for 8 weeks in both clinics (clinic SBP reduction: nifedipine GITS vs amlodipine = 14.4  vs 14.5 mmHg, *P* = .91; clinic DBP reduction: nifedipine GITS vs amlodipine = 6.7  vs 7.5 mmHg, *P* = .24) and ambulatory (24‐hour SBP reduction: nifedipine GITS vs amlodipine = 10.9  vs 10.3 mmHg, *P* = .56; 24‐hour DBP reduction: nifedipine‐GITS vs amlodipine = 6.3  vs 6.5 mmHg, *P* = .83) BP measurement between the nifedipine GITS group (n = 248) and amlodipine groups (n = 257). Furthermore, the incidence of adverse events was similar between the two groups. However, after missing a dose of medication, greater reduction in 24‐hour DBP (nifedipine GITS vs amlodipine = ‐4.3  vs ‐5.5 mmHg, *P* = .04) and daytime DBP (nifedipine GITS vs amlodipine = ‐4.5 mmHg vs. ‐6.0 mmHg, *P* = .02) was observed in the amlodipine group, indicating that when a dose is delayed for hours on monotherapy, the efficacy of nifedipine GITS might be reduced markedly.^13^

Recently, another RCT compared the average nighttime SBP reduction achieved with nifedipine GITS (n = 49) and amlodipine (n = 49), focusing on young and middle‐aged adults (18–65 years) with nondipper hypertension. After 8 weeks of treatment, no difference was observed between these two agents with respect to nighttime SBP reduction (nifedipine‐GITS vs amlodipine = −10.8  vs −10.5 mmHg, *P* = .898) and dipping rhythm restoration and arterial elasticity improvement, irrespective of daytime (−11.5  vs −10.9 mmHg, *P* > .05) or nighttime administration (−9.9  vs −9.9 mmHg, *P* > .05).^14^, ^25^

Thus, short‐acting nifedipine with extended‐release formulation appeared to demonstrate a BP‐lowering effect similar to that of long‐acting CCB (amlodipine), despite the fact that when a dose of medication was missed, the latter seemed to be more efficacious.^21^, ^22^, ^23^

Given the similar efficacies of the generic and the brand‐name antihypertensive medications, and of the extended‐release formulations of short‐acting CCB and long‐acting CCB, we further compared the clinical outcomes of the generic nifedipine SRFC CYH and of the brand‐name amlodipine Norvasc, and observed no significant differences.

### 4.4 Study limitations

The present study has some limitations. First, the sample size is relatively small. Further studies with large sample size and longer follow‐up period are indicated in the future. Second, the study was conducted at a single medical center in Taiwan. Patients in the medical center might receive better medical care. Due to potential differences between the setting of the institute and that of other institutes and between the health insurance cover of the patients at this institute and those at other institutes and hospitals, further studies are needed to confirm whether the results could be applied on a global scale. Third, we do not have the information about the duration of hypertension in each participant, which might be a possible confounding factor. Fourth, the data on the comorbidities and clinical outcomes of the patients were extracted from the Taipei Veterans General Hospital database. However, there may be a potential risk of ascertainment bias. However, the same methodologies have been applied using the National Health Insurance Research Database in Taiwan, which has been verified in numerous studies.^26^, ^27^, ^28^, ^29^, ^30^ Fifth, this study may have been subject to selection bias since the patients who were prescribed nifedipine generally had more comorbidities. Although rigorous propensity score matching was conducted to balance potential differences between the treatment groups, potential selection bias and unmeasured confounding factors may have affected the results. Finally, given the retrospective design of the present study, our results can lay sufficient grounds for the development of a hypothesis, which should then be validated by conducting well‐powered RCTs with sufficient follow‐up.

## 5 CONCLUSION

The generic nifedipine SRFC CYH was comparable to the brand name Norvasc in terms of CV death, nonfatal MI, nonfatal ischemic stroke, heart failure hospitalization, and composite outcomes.

## CONFLICT OF INTEREST

None.

## AUTHOR CONTRIBUTIONS

HWL contributed to conception and design, interpretation of data, and drafted the manuscript. CCH contributed to conception, data acquisition, analysis and interpretation of data, drafted and critically revised the manuscript. HBL contributed to conception and design, data acquisition, and drafted the manuscript. YJL contributed to conception and design, data acquisition, and drafted the manuscript. All authors gave final approval and agreed to be accountable for all aspects of work ensuring integrity and accuracy.
